# Choosing sensitivity analyses for randomised trials: principles

**DOI:** 10.1186/1471-2288-14-11

**Published:** 2014-01-24

**Authors:** Tim P Morris, Brennan C Kahan, Ian R White

**Affiliations:** 1 Hub for Trials Methodology Research, MRC Clinical Trials Unit at UCL, Aviation House, 125 Kingsway, London WC2B 6NH, UK; 2 Pragmatic Clinical Trials Unit, Queen Mary University of London, 58 Turner Street, London E1 2AB, UK; 3 MRC Biostatistics Unit, Cambridge Institute of Public Health, Forvie Site, Robinson Way, Cambridge Biomedical Campus, Cambridge CB2 0SR, UK

**Keywords:** Sensitivity analysis, Randomised trials, Clinical trials, RCT, Missing data

## Abstract

**Background:**

Sensitivity analyses are an important tool for understanding the extent to which the results of randomised trials depend upon the assumptions of the analysis. There is currently no guidance governing the choice of sensitivity analyses.

**Discussion:**

We provide a principled approach to choosing sensitivity analyses through the consideration of the following questions: 1) Does the proposed sensitivity analysis address the same question as the primary analysis? 2) Is it possible for the proposed sensitivity analysis to return a different result to the primary analysis? 3) If the results do differ, is there any uncertainty as to which will be believed? Answering all of these questions in the affirmative will help researchers to identify relevant sensitivity analyses. Treating analyses as sensitivity analyses when one or more of the answers are negative can be misleading and confuse the interpretation of studies. The value of these questions is illustrated with several examples.

**Summary:**

By removing unreasonable analyses that might have been performed, these questions will lead to relevant sensitivity analyses, which help to assess the robustness of trial results.

## Background

In randomised trials researchers are required to specify analyses before seeing the outcome data, to select one of these as the primary analysis, (ideally) to make this plan public, and to adhere to the specified analyses on receipt of the outcome data. There are two reasons for this approach. First, it prevents researchers from cherry picking their favoured analyses after seeing the data. Second, choosing the method for the primary analysis based on trial data has been shown to lead to unreliable results in several settings [1-4].

Pre-specified analyses make assumptions that may be strong, unverifiable, or not supported by the data. ‘Sensitivity analysis’ aims to investigate whether the results of important analyses are sensitive or robust to violations of the assumptions by performing analyses addressing a specific clinical question under contrasting assumptions. Despite articles advocating sensitivity analysis [5], there are currently no principles governing how relevant sensitivity analyses should be chosen. The following three questions should be considered to determine whether a sensitivity analysis is worthwhile: 

1. Does the proposed sensitivity analysis address the same question as the primary analysis?

2. Is it possible for the proposed sensitivity analysis to arrive at a different conclusion to the primary analysis?

3. If the proposed sensitivity analysis leads to a different conclusion to the primary analysis, is there a genuine degree of uncertainty as to which will be believed?

To qualify as a sensitivity analysis, the answer to all of the above questions should be *yes*. Analyses that address different questions to the primary analysis may be important, but should be classified as secondary, not sensitivity analyses; considering them as sensitivity analyses may lead to false anxiety about the robustness of results. Analyses that will always lead to the same conclusion as the primary analysis are dangerous, as they falsely reassure us about the robustness of results. If there is no uncertainty as to which analysis is more believable, the analysis that would not be believed should be dropped.

**Figure 1:**
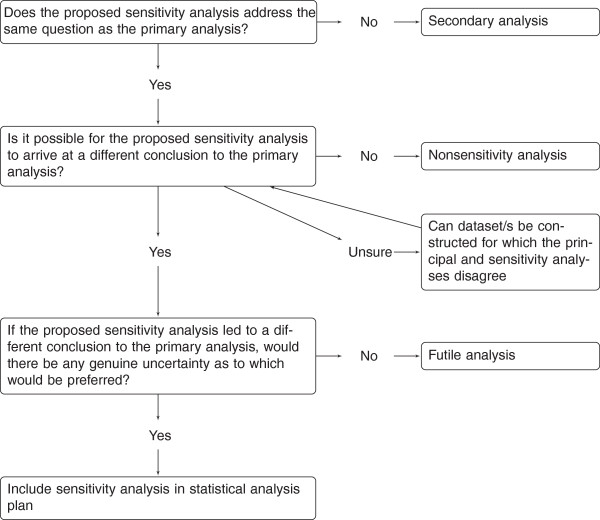
**Questions for a sensitivity analysis.** Schematic for deciding whether to proceed with the proposed sensitivity analysis.

Below, we expand on the reasons for asking each question, and demonstrate their usage with some real examples. Because we are advocating the use of these questions at the stage of writing the statistical analysis plan, we do not consider the results of our examples, but whether the sensitivity analysis described is worthwhile. Figure 1 is provided as a quick reference tool.

## Discussion

### Does the proposed sensitivity analysis address the same question as the primary analysis?

It would seem very strange to consider how sensitive an answer is to the question being asked. We should be looking at how sensitive an answer is to asking the question in a different way. If two seemingly similar analyses address different questions, the proposed sensitivity analysis is correctly regarded as a secondary analysis (note that we use ‘secondary’ to refer to a secondary analysis rather than the analysis of a secondary outcome).

#### Example

The Multicentre Aneurysm Screening Study group randomised 67,800 men to receive an invitation to an abdominal ultrasound scan or not [6]. Of those invited to receive an abdominal scan, 20% did not accept. The primary analysis was by intention to treat, thus estimating the effect of being randomised to abdominal ultrasound. Another analysis investigated the complier average causal effect, which considers what the (average) effect of treatment was in patients who would have adhered to protocol however they were randomised [7]. These questions are different, and observing different results should not shake our confidence in either. The CACE analysis was a secondary analysis, not a sensitivity analysis.

It is common for authors to compare the results of intention-to-treat with per-protocol analysis; see for example [8,9]. While it is hard to pin down the precise question of per-protocol analysis [10], this is clearly different to the question intention-to-treat addresses. Per-protocol analysis should not therefore be considered as a sensitivity analysis for intention-to-treat but as a secondary analysis, if at all.

### Is it possible for the proposed sensitivity analysis to arrive at a different conclusion to the primary analysis?

This question aims to check whether an alternative analysis can be considered a sensitivity analysis or if it is a foregone conclusion. Performing two different analyses does not mean they can lead to different results. If they cannot, the analysis is misguided and can tell us nothing about the sensitivity of our conclusions.

If the answer to this question is unclear at the stage of writing the statistical analysis plan, a helpful exercise is to attempt to construct one or more datasets in which the primary and sensitivity analysis disagree. If sensitivity analyses are motivated by concerns about certain features of the data, this should not be difficult.

#### Example

Consider the proposal for handling missing data in Table two of Thabane et al [5]. The suggestion is to ‘analyse only complete cases’ and then ‘impute the missing data …and redo the analysis’. This will not necessarily assess how robust the results are to the missing data, as demonstrated by the following example.

In a protocol for an ongoing study, Zheng et al. describe a randomised trial aiming to assess the effect of Baduanjin exercise on physical and mental health in 222 college students [11]. The primary analysis will compare the mean lumbar muscle strength using a *t*-test. It is anticipated that outcomes will be missing for some participants. (The following is our illustration, and not described by the authors.) Assume that the primary analysis is in complete cases only, but the investigators wish to investigate how sensitive results are to dropping participants with missing data. They decide to use multiple imputation, where the model for imputation assumes lumbar muscle strength is normally distributed with different means but equal variances in the two treatment groups.

The imputation model described makes identical assumptions to the *t*-test and, with a sufficient number of imputations, the multiple imputation analysis will give near identical results. Simply imputing data does not necessarily make different assumptions. The fact that the results of two analyses are almost identical should not be reassuring: this is equivalent to being reassured that running one analysis twice gives the same results. Running two analyses which make identical assumptions gives us false confidence in the robustness of results. Note that this analysis may have value as a check that multiple imputation is working correctly, but only as a springboard to other imputation approaches.

### Is there a genuine degree of uncertainty as to which analysis will be believed?

Assume the proposed sensitivity analysis addresses the same question as the primary analysis and can lead to different conclusions. It should then be considered whether either analysis is obviously and always to be preferred. If it is clear that one analysis would always be believed over the other, the former should be the primary analysis and the latter should not be done. Sensitivity analysis is not an opportunity to perform an unreasonable analysis.

A sensitivity analysis should be derived from assumptions that seem plausible. With respect to the trial design, particularly strong, questionable or untestable assumptions made by the primary analysis should, where possible, be addressed by one or more alternative analyses that make different assumptions. Given the trial design and assumptions, the sensitivity analysis should be asymptotically unbiased for estimation of the treatment effect, and control the rate of type I errors, and thus coverage. The sensitivity analysis may be less powerful than the primary analysis.

#### Example

Returning to the missing data example above, if Zheng et al. were to use single (as opposed to multiple) imputation as a sensitivity analysis, as suggested in [5], this could well lead to different conclusions, despite our criticism above that the model for imputation is identical to the model for the primary analysis. Single imputation fails to allow for the uncertainty induced by missing data, and does not in general lead to valid inferences [12]. The estimated standard error of the treatment effect will be too small, implying inflation of type I error rates and under-coverage of confidence intervals. The analysis should thus not undermine the primary analysis of covariance.

Similarly, performing ‘one analysis that accounts for clustering and one that ignores it’, as proposed in [5], may be unwise. In general an analysis that accounts for clustering is used because clustering arises through the design [13]. If a cluster-randomised trial were primarily analysed allowing for the clustering, but a subsequent analysis ignoring the clustering led to different conclusions, there would be no degree of uncertainty as to which we believe. However, the best approach to account for clustering may be unclear. Hu et al. consider approaches to analysing a study with a longitudinal binary outcome [14], comparing random effects approaches with generalised estimating equations. This is reasonable because there tends to be some uncertainty as to which method is preferred.

#### Caveat: practical constraints

We note that there may be settings where exceptions to the third question are made for unavoidable reasons. For example, constraints on time, reliance on a methodology which is not well understood at the stage of writing, and a lack of software with the ability to run the analysis may make the ‘preferred analysis’ impractical for certain cases. This may happen in exceptional cases.

#### Example

The Paramedic trial is designed to assess whether survival of cardiac arrest patients can be improved by equipping ambulances with a mechanical chest compression device, compared to manual chest compressions by the crew [15]. Ambulances are randomised in a 1:2 ratio. Ambulances may move between different sites, and the crew may move between ambulances and sites, meaning the data involves cross-classified clustering. The principal analysis for this trial accounts for ambulance, but not for crew members, or the site the ambulance left from or returned to. Although an analysis which fully accounts for all three types of clustering may be preferred in theory, fitting such a model may be difficult, and might require the development of new methods and software.

We expect this issue to be rare. It arises because one analysis is preferred in theory but not in practice, and so the answer the question 3 can in fact be regarded as *no*.

### Examples

Further examples of proposed sensitivity analyses which our questions regard as reasonable are given below.

#### Missing data assumptions

Consider again the trial described by Zheng et al [11]. Recall that the continuous outcome is anticipated to be missing for some participants. Suppose the primary analysis assumes data are *missing at random* and multiply imputes lumbar muscle strength separately for the two treatment groups, using an imputation model that also conditions on secondary (*auxiliary*) outcomes such as physical fitness, stress and quality of life. This primary analysis is valid under a *missing at random* assumption, and may give different results to analysis of the complete cases. However the assumption that missing values are missing at random is untestable. What if the data are truly *missing not at random*? It is considered plausible that lumbar muscle strength is less likely to be observed in individuals with lower values, and so missing values might on average be one unit lower than the observed outcomes of otherwise comparable individuals. Our sensitivity analysis might then be to *subtract one* from every imputed value and re-analyse the imputed data. Further sensitivity analyses could assume that this mechanism only occurred within one treatment group, that it was stronger than *subtract one*, or that it was in the opposite direction (*add one*) [16].

The sensitivity analyses are reasonable because they: 1) address the same question as the primary analysis, under different assumptions; 2) may or may not lead to different conclusions; and 3) involve different assumptions which may be plausible, although some may be less so: there is genuine uncertainty about the most plausible assumption [16].

#### Definitions of outcome

In a randomised trial in neutropenic patients, de Pauw et al. considered the effect of antifungal therapy on outcome [17]. The primary outcome was defined by a five-part composite endpoint, one part being ‘fever resolution’. Because fever resolution was hard to define, sensitivity analyses included using alternative definitions and feeding these into the five-part composite endpoint.

The Copers trial [18] is designed to evaluate a self-management course for patients with chronic musculoskeletal pain. The primary outcome is the mean of of three questions about Q1) the amount of pain-related disability the participant is currently experiencing, and whether the participant’s ability to Q2) work, and Q3) interact socially, has changed. Each is scored out of 10 with a high score intended to identify a negative outcome for all three questions. However, there is concern that for Q2 and Q3 some participants may be confused about whether a high or low score indicates a negative outcome. A planned sensitivity analysis is thus to redefine participants’ answers so that anyone with a score of two or lower for Q1, but eight or higher for Q2 or Q3 has their scoring reversed for Q2 and/or Q3. The primary analysis is then repeated with this definition of outcome.

Ideally, outcome variables would be unambiguously defined, but this is not always the case. For both of the above examples we regard the answer to our three questions as ‘yes’. The same question is being addressed, but assumptions about the definition of outcome are different, and it is not certain that one definition is correct.

## Summary

Sensitivity analyses should be carefully chosen and predefined as far as possible, where the proposed analyses involve a range of plausible and contrasting assumptions. Some analyses should rightly be given more emphasis than others, and the primary analysis should be the one that seems to carry the most weight prior to seeing the data. Our three questions help to identify whether an alternative analysis is reasonable as a sensitivity analysis.

It is the need to prespecify analyses that makes these questions particularly useful in the context of randomised trials. However, they are also helpful with observational studies and when applied post-hoc.

Through discussions with trial statisticians we believe sensitivity analyses are used widely, but often informally, and results are largely unreported. If the screening questions outlined above are considered at the stage of writing the statistical analysis plan, sensitivity analyses will be relevant to the clinical question, and the number to perform and report will be reduced. We hope that in future they will be sensibly chosen and more widely reported.

## Competing interests

The authors declare that they have no competing interests.

## Authors’ contributions

TPM and BCK had the original idea for the article. TPM drafted the article. IRW added further ideas. All authors read and approved the submitted manuscript.

## Pre-publication history

The pre-publication history for this paper can be accessed here:

http://www.biomedcentral.com/1471-2288/14/11/prepub
